# Care and Treatment of Epileptics

**Published:** 1900-02

**Authors:** 


### Book Reviews.

CARE AND TREATMENT OF EPILEPTICS. By WILLIAM PRYOR LETCHWORTH, LL. D., Ex-President of the New York State Board of Charities; Ex-President of the Eleventh National Conference of Charities and Correction; Author of The Insane in Foreign Countries, Child of the State, Relief and Reform, Homes for Homeless Children, etc. Illustrated. Octavo, pp. 246. New York and London: G. P. Putnam's Sons, The Knickerbocker Press. 1900.

Mr. Letchworth has long been known to this community as a scientific student of public institutions. His work on the care of the insane was a presentation of that subject as seen by him in Europe and America.

The care and treatment of epileptics is, first, a historical review of what public philantrophy has done in America and in Europe for this afflicted class. It is second, a plea for more and greater effort in the work of rescuing the epileptic from almshouses and homes for the feeble-minded; and third it is a descriptive work of every public institution in this country and of the more prominent ones in England and the continent.

The first state to act in behalf of epileptics was Ohio. In 1891 was laid the corner stone of the Ohio Asylum for epileptics and epileptic insane. In this colony, great attention has always been paid to diet, to education, to daily occupation and to amusements. In five years there were 1,295 patients, of these 81 were discharged cured.

The chapter on the Craig colony is naturally of great interest to us. Mr. Letchworth has given a most careful description of the buildings, the sanitary rules which have been adopted, and the general management of the various departments. No one can read this chapter without realising that the state of New York has most nobly met its obligation. Every one must feel that the board of managers has done its work most carefully and conscientiously.

The cottage plan and its advantages are set forth. The dietary is given in detail, as much stress is laid, and rightly so, upon the important rôle which this plays in the treatment of epilepsy. The advantages accruing to the epileptic from work adapted to individual needs, and from education are clearly pointed out. Facts and not theories are given.

We quote the following record respecting the first fifty patients admitted and their condition at the end of five months of colony life: Total number of seizures, first month, 708; fifth month, 315. Average individual seizures, first month, 14; fifth month, 6.

This to our mind represents what a proper environment can do for those epileptics who come from unfavorable surroundings.

Craig Colony is much too small to accommodate the needy and worthy epileptics of New York. Its present capacity is only 360. When the new group of buildings is completed it will only accommodate 620 patients, but it is a grand beginning and is certainly bound to be for all time a great and beneficent charity and an institution for scientific research.

Massachusetts, New Jersey and Texas have each provided institutions exclusively for epilpetics. Other states have provided for epileptics and the feeble-minded in the same institutions. This, as Mr. Letchworth clearly shows, is not only an error, but is a position wrong to the epileptic. If this book does nothing else it must convince all students of this subject that the poor epileptics at any rate must be protected and cared for in specially conducted institutions. Their care must include medical, pedagogic and industrial work.

This book is most beautifully printed and illustrated. It is a strong argument for the establishment of such institutions as Craig Colony in every state in the Union. It is a splendid guide to those who would build and manage such institutions. Above all, it is a noble monument to the self-sacrificing, devoted author, who has given the greater portion of his life to relieving his less fortunate dependent brothers. J. W. P.

